# Differential Immune Responses and Underlying Mechanisms of Metabolic Reprogramming in Smooth and Rough Variants of *Mycobacterium peregrinum* Infections

**DOI:** 10.3390/pathogens12121446

**Published:** 2023-12-13

**Authors:** Ji Won Lee, Ho Won Kim, A-Reum Yu, Hoe Sun Yoon, Minji Kang, Hwan-Woo Park, Sung Ki Lee, Jake Whang, Jong-Seok Kim

**Affiliations:** 1 Myunggok Medical Research Institute, College of Medicine, Konyang University, Daejeon 35365, Republic of Korea; smileday0103@naver.com (J.W.L.); kimong104@naver.com (H.W.K.); kyoaor22@hanmail.net (A.-R.Y.); cordelia_sun@naver.com (H.S.Y.); 2 Korea Mycobacterium Resource Center (KMRC), Department of Research and Development, The Korean Institute of Tuberculosis, Osong 28158, Republic of Korea; mjkangs92@naver.com; 3 Department of Cell Biology, College of Medicine, Konyang University, Daejeon 35365, Republic of Korea; hwanwoopark@konyang.ac.kr; 4 Department of Obstetrics and Gynecology, Konyang University Hospital, Daejeon 35365, Republic of Korea; sklee@kyuh.ac.kr

**Keywords:** *Mycobacterium peregrinum*, immune response, inflammatory cytokine, metabolic reprogramming

## Abstract

*Mycobacterium peregrinum* (*Mpgm*) is a rapidly growing mycobacteria that is classified as a nontuberculous mycobacterium (NTM) and is commonly found in environmental sources such as soil, water, and animals. *Mpgm* is considered an opportunistic pathogen that causes infection in immunocompromised individuals or those with underlying medical conditions. Although there have been clinical reports on *Mpgm*, reports of the immune response and metabolic reprogramming have not been published. Thus, we studied standard *Mpgm*-ATCC and two clinical strains (*Mpgm*-S and *Mpgm*-R) using macrophages and mouse bone marrow-derived cells. *Mpgm* has two types of colony morphologies: smooth and rough. We grew all strains on the 7H10 agar medium to visually validate the morphology. Cytokine levels were measured via ELISA and real-time PCR. The changes in mitochondrial function and glycolysis in *Mpgm*-infected macrophages were measured using an extracellular flux analyzer. *Mpgm*-S-infected macrophages showed elevated levels of inflammatory cytokines, including interleukin (IL)-6, IL-12p40, and tumor necrosis factor (TNF)-α, compared to *Mpgm*-ATCC- and *Mpgm*-R-infected macrophages. Additionally, our findings revealed metabolic changes in *Mpgm*-ATCC and two clinical strains (*Mpgm*-S and *Mpgm*-R) during infection; significant changes were observed in the mitochondrial respiration, extracellular acidification, and the oxygen consumption of BMDMs upon *Mpgm*-S infection. In summary, within the strains examined, *Mpgm*-S displayed greater virulence, triggered a heightened immune response, and induced more profound shifts in bioenergetic metabolism than *Mpgm*-ATCC and *Mpgm*-R. This study is the first to document distinct immune responses and metabolic reorganization following *Mpgm* infection. These findings lay a crucial foundation for further investigations into the pathogenesis of *Mpgm.*

## 1. Introduction

Nontuberculous mycobacteria (NTM) include a variety of Mycobacterium species that do not include the obligate pathogens *Mycobacterium tuberculosis* complex and *Mycobacterium leprae* [1]. Until 1950, these organisms were generally regarded as nonpathogenic due to their limited virulence [2,3]. However, NTMs have now emerged as significant human pathogens, causing widespread clinical disease in individuals with weakened immune systems and those with underlying medical conditions. NTMs can be found throughout the environment, and specific species, such as *Mycobacterium avium* and *Mycobacterium abscessus*, are responsible for causing opportunistic infections in humans [4].

*Mycobacterium peregrinum* (*Mpgm*) is a rapidly growing NTM (RGM). These mycobacteria are typically categorized into distinct groups, which include the *Mycobacterium fortuitum* group, the *Mycobacterium chelonae abscessus* group, and the *Mycobacterium mucogenicum* group. Specifically, *Mpgm* is in the *Mycobacterium fortuitum* group and contributes to approximately 2% of all RGM infections [5].

*Mpgm* has been increasingly recognized as a pathogen in both immunocompetent and immunocompromised individuals [6]. A review of 19 publications sourced from the PubMed and MEDLINE databases revealed a total of 21 cases involving *Mpgm* infection. To summarize, the most frequently affected sites were the skin and soft tissues, and infections were often associated with surgical or artificial device-related incidents. Additionally, cases of catheter-related bloodstream infection, pneumonia, lymphadenitis, tonsillar abscess, and infective endocarditis attributable to *Mpgm* were also reported. Nine cases (42.9%) were identified in immunocompromised individuals. Notably, *Mpgm* induced sporadic invasive infections even in immunocompetent patients. Furthermore, pneumonia caused by *Mpgm* was reported in two previously healthy patients (2/4, 50%) [7]. Despite its clinical significance, there is limited understanding of the host immune response to *Mpgm* infection, and no studies have investigated in vitro differential immune responses based on morphological analysis. The aim of our research was to explore for the differences between immune responses, cytokine expression, signaling pathways, and changes in metabolic flux when bone marrow-derived macrophages (BMDMs) are infected with *Mpgm* for the first time.

In this study, we examined the immune responses and metabolic changes to three different strains, including the standard ATCC 14467 strain (*Mpgm*-ATCC) and clinical strains, i.e., the designated S-type (*Mpgm*-S; KMRC-A9381) and R-type (*Mpgm*-R; KMRC B0585). We investigated the immune response because mycobacteria exhibit the capacity to persist and multiply as intracellular pathogens within macrophages. Furthermore, we assessed cell survival and cytotoxicity following infection with *Mpgm*. Moreover, we conducted a comparative analysis of cytokine expression patterns and examined the phosphorylation of MAPK (mitogen-activated protein kinases) and NF-κB (nuclear factor kappa-light-chain-enhancer of activated B cells) in response to macrophage activation induced by each strain. Finally, we performed extracellular flux analysis to determine whether energy metabolism regulation is affected in BMDMs upon infection with *Mpgm* strains.

## 2. Materials and Methods

### 2.1. Bacterial Conditions and Growth

The *Mycobacterium peregrinum* reference strain (ATCC 14467) was acquired from the American Type Culture Collection (ATCC, Manassas, VA, USA). The two clinical strains (KMRC-A9381 and KMRC-B0585) were obtained from The Korean Mycobacterium Resource Center (KMRC) of the Korean Institute of Tuberculosis (Osong, Republic of Korea). These strains were cultured in Middlebrook 7H9 Broth medium (Difco Laboratories, Detroit, MI, USA) supplemented with 10% oleic albumin dextrose catalase (OADC: Becton Dickinson, Sparks, MD, USA) for 5–7 days at 37 °C.

### 2.2. Preparation of Single-Cell Bacterial Stocks

*Mpgm* was cultured in 50 mL tubes containing the 7H9 broth medium. The tube was placed in a shaking incubator and cultured at 37 °C with shaking at 160–180 rpm. After growing for approximately 5 to 7 days, 100 μL of the culture medium was transferred to a 96-well plate, and the OD_600_ value was measured. When the OD_600_ value reached 0.8–0.9, the culture was scaled up by sequentially increasing the volume from a 125 mL flask to a 1 L flask. Once the OD_600_ value of the 1 L flask culture exceeded 0.9, preparations were made to create a stock. The 1 L culture solution was dispensed into a 50 mL conical tube, and the tube was centrifuged at 4 °C and 3000 rpm for 30 min. After removing the supernatant, PBS was added to the remaining pellet and the sample was mixed using a vortex mixer. This process was repeated, and after the pellets were collected in one tube, they were dissolved in PBS and filtered through a strainer. The filtered solution was centrifuged at 3000× *g* for 30 min at 4 °C. After removing the supernatant, the pellet was suspended in a storage medium containing 7H9 powder, 50% glycerol, 10% OADC, and 20% Tween 80. The bacterial suspension was loaded into a 20 mL syringe and filtered through a 5 μm syringe filter. 50 μL of the filtered bacteria were aliquoted and stored at −80 °C.

### 2.3. Bone Marrow-Derived Macrophage Culture and Ethics Statement

Bone marrow-derived macrophages (BMDMs) were obtained from the femurs and tibias of female C57BL/6 mice aged 6–8 weeks (DBL, ChungcheongBuk-do, Republic of Korea). The cells were harvested and then centrifuged at 1200 rpm for 3 min. The pellet was resuspended in Dulbecco’s modified Eagle’s medium (DMEM; Welgene Co.; Daegu, Republic of Korea) containing glutamine, 1× penicillin–streptomycin (Welgene Co.; Daegu, Republic of Korea), 10% fetal bovine serum (FBS; Welgene Co.; Daegu, Republic of Korea), and 10 ng/mL recombinant murine M-CSF (PeproTech, Cranbury, NJ, USA). On day three, the media were supplemented, and the culture was continued for an additional three days. Following a total of six days of culture, nonadherent cells were removed, and the differentiated macrophages were detached via incubation with trypsin−0.25% EDTA (Welgene Co.; Daegu, Republic of Korea) for 60 s in an incubator. The detached cells were centrifuged at 1200 rpm for 3 min, resuspended, and seeded in complete DMEM.

The animals were housed in an SPF barrier room under controlled conditions on a 12 h light–dark cycle and a constant temperature (25 °C). The experiments were performed in accordance with the Animal Care and Guiding Principles for Animal Experiments and were approved by the University of Konyang Animal Care and Use Committee (21-07-E-01).

### 2.4. Thin-Layer Chromatography

The extraction and identification of bacterial total lipids and glycopeptidolipids (GPLs) were performed according to established protocols [8]. To isolate total lipids from *Mpgm*, a chloroform/methanol mixture (2:1, *v*/*v*) was used, and the mixture was sonicated for 20 min. The resulting phase was separated via centrifugation. The total lipid fraction was analyzed by two-dimensional thin-layer chromatography (2D-TLC) after dotting at a concentration of 400 μg/μL on a TLC plate. The purified lipids were separated with chloroform/methanol/acetone/acetic acid (90:10:6:1, *v*/*v*/*v*/*v*) and then with chloroform/methanol/water (90:10:1, *v*/*v*/*v*). The TLC plate was visualized by spraying with a 10% sulfuric acid (H_2_SO_4_) solution and subsequently heating at 200 °C for 10 min.

### 2.5. Antimicrobial Susceptibility Test

Bacterial cultures were inoculated into growth media containing different concentrations of antibiotics, and then bacterial growth was visually monitored by the naked eye for a specified period of time. Primary determination of antibiotic susceptibility was performed on day 3 of culture by assessing whether noticeable bacterial growth occurred in each well. Additional readings were performed on days 7, 10, and 14 to investigate the development of antibiotic resistance caused by clarithromycin. During this time, we assessed the presence or absence of bacterial growth in the wells and established the minimum inhibitory concentration (MIC), which is the lowest concentration at which bacterial growth is inhibited.

### 2.6. Intracellular Staining (Confocal Microscopy)

The initial bacterial concentration of 1 × 10^8^ cells/mL was utilized and the bacteria were washed once with phosphate-buffered saline (PBS) and subsequently diluted in 500 μL of PBS. They were then coupled with 10 μM carboxyfluorescein diacetate succinimidyl ester (CFSE; C34554, Invitrogen, Carlsbad, CA, USA) and incubated for 2 h at room temperature in the dark. After incubation, the bacteria were washed twice with PBS supplemented with 5% FBS, and subjected to bath sonication five times for 3 s; the samples were vortexed gently to resuspend the bacteria. CFSE-labeled bacteria were used to infect BMDMs at an MOI of 5 for 4 h. After infection, the cells were washed twice and fixed with 3.7% formaldehyde for 20 min. Cell permeabilization was performed using 0.2% Triton-X 100 for 10 min, and blocking was achieved using 3% BSA for 1 h at room temperature. Stained cortical F-actin was detected using phalloidin-Texas red (Invitrogen) staining, and nuclei were stained using DAPI (Sigma, St Louis, MO, USA). The cells were visualized by fluorescence microscopy using a laser scanning confocal microscope (Carl Zeiss, Jena, Germany).

### 2.7. Phagocytosis Assay and Infection Kinetics of M. peregrinum

BMDMs were seeded at 1.5 × 10^5^ cells per 48-well plate. After 24 h, BMDMs were infected with *Mpgm* using a multiplicity of infection (MOI) of 1 for 1, 2, 4, or 8 h or at MOIs of 1, 5, or 10 for 4 h at 37 °C. After infection, the cells were washed three times with Dulbecco’s phosphate-buffered saline (DPBS; Welgene Co.; Daegu, Republic of Korea) and treated with 20 μg/mL amikacin (Sigma, St. Louis, MO, USA) to remove all extracellular bacteria. After 1 h, the amikacin-treated supernatant was removed, and the cells were washed three times with DPBS. The culture supernatants were aspirated, and the cells were lysed using 0.05% Triton X-100 (Sigma). Then, the lysates were plated in tenfold serial dilutions onto 7H10 agar to quantify the number of viable bacteria. Colonies were counted after 4 days of incubation at 37 °C.

The infection kinetics of *Mpgm* were evaluated in a method similar to the experimental protocol described above. BMDMs were seeded at 1.5 × 10^5^ cells per 48-well plate. After 24 h, BMDMs were infected with *Mpgm* at an MOI of 1 for 4 h at 37 °C and further cultivated in a fresh complete medium for 3 days. On days 0, 1, 2, and 3 after infection, the culture supernatants were removed and the cells were lysed. The lysates were then subjected to tenfold serial dilutions and plated onto 7H10 agar. After 4 days of incubation, the colonies were counted. The results of these experiments were reported as the mean CFU ± standard deviation (SD) per 1.5 × 10^5^ cells.

### 2.8. Cell Viability Assay and Cytotoxicity Assay

Cell viability was determined using Maestro Z (Axion Biosystems, Atlanta, GA, USA). Before cell seeding, a baseline was measured with the culture medium. A total of 5 × 10^4^ cells per well were seeded in 96-well plates and incubated overnight. After incubation, the cells were washed twice with DPBS and infected with *Mpgm* at MOIs of 1, 2, or 5 for 24 h. The acquired data were analyzed using dedicated software to quantify cell viability changes over time. The Maestro Z quantification of cell viability uses impedance. Impedance is the principle by which electrical signals are transmitted to electrodes, and the 96-well cytoview-Z plate has electrodes built into it, which allows for electrical signals to be recognized. Impedance measures the degree to which electrical signals are blocked by the electrode and cell interface. When a cell attaches, the electrical signal is blocked and detected as an increase in impedance, and when a cell dies, it is detected as a decrease in impedance. The results of these experiments were reported as impedance values ± standard deviation (SD) per 5 × 10^4^ cells.

Cellular cytotoxicity was measured using an LDH cytotoxicity assay kit (DoGenBIO, Seoul, Republic of Korea) according to the manufacturer’s protocol. A total of 5 × 10^4^ cells per well were seeded into 96-well plates overnight in DMEM. The next day, the cells were washed with DPBS and infected with *Mpgm* at MOIs of 1, 2, or 5 for 24 h. After 24 h of incubation, 10 µL of the supernatant was transferred into a 96-well plate, and 100 µL of Dye Solution was added. For the quantification of the maximum LDH level, the high control underwent treatment with lysis solution at 37 °C for 5 min, followed by centrifugation at 1200 rpm for 3 min. The resulting supernatant was subsequently used for analysis. The plate was incubated for 1 h in the dark, and absorbance was measured at 450 nm using a microplate reader (Epoch, BioTek, Winooski, VT, USA). Percent cytotoxicity was determined by calculating the difference between the LDH release at a high control and the LDH release at a low control. This difference was then divided by the difference between the LDH release in the experimental conditions and the low control, and the result was multiplied by 100 to express cytotoxicity as a percentage.

### 2.9. Measurement of Cytokines via Enzyme-Linked Immunosorbent Assay (ELISA)

A total of 1.5 × 10^5^ cells per well were seeded in 48-well plates. Culture media from the infected BMDMs at MOIs of 1, 2, or 5, and noninfected BMDMs were collected at 24 h post infection. The collected supernatant was centrifuged at 1200 rpm for 3 min. Samples were preserved at −80 °C until analysis. In the assessment of IL-6, IL-10, IL-12p40, and TNF-α, the conditioned medium was analyzed using OptEIA ELISA kits (BD Biosciences, San Diego, CA, USA) according to the manufacturers’ guidelines. Ninety-six-well plates were incubated overnight at 4 °C with 100 µL/well of capture antibody in a coating buffer. After three washes with PBS containing 0.05% Tween-20 (LPS solution, Daejeon, Republic of Korea), the wells were blocked with 200 µL of ELISA/ELISPOT Diluent (1×) for 1 h. Subsequently, 100 µL of the samples and standards was added to each well, and the plate was incubated for 2 h at room temperature. Following this incubation, the wells underwent 30 min incubation at room temperature in the dark with detection antibody and streptavidin-HRP in an ELISA/ELISPOT diluent (1×). The plates were then treated with a TMB solution (1×) for 30 min, and the reaction was halted by the addition of a Stop solution. The optical density absorbance at 450 nm was measured using a microplate spectrophotometer (BioTek), and the values were calculated based on the standard curve.

### 2.10. RNA Extraction and Real-Time PCR

A total of 1 × 10^6^ cells per well were seeded in 6-well plates. Total RNA was extracted from BMDMs infected with *Mpgm* strains at MOIs of 5 and noninfected BMDMs at 24 h postinfection. RNA was isolated using an AccuPrep Universal RNA extraction kit (BI-ONEER, Daejeon, Republic of Korea). cDNA was synthesized using a PrimeScript First Strand cDNA Synthesis Kit (Takara, Tokyo, Japan). The obtained cDNA was analyzed for the expression of genes including β-actin, interleukin (IL)-6, IL-10, IL-12p40, and TNF-α. Quantitative real-time PCR was performed using cDNA, BioFACT™ 2X Real-Time PCR Master Mix, including SYBR^®^ Green I in mixture (BioFACT, Daejeon, Republic of Korea), and specific primers. The following primer pairs were used in the analysis: mouse β-actin, 5′-TACCCAGGCATTGCTGACA GG-3′ and 5′-ACTTGCGGTGCACGATGGA-3′; mouse IL-6, 5′-GATGGATGCTACCAAACTGGAT-3′ and 5′-CCAGGTAGCTATGGTAC TCCAGA-3′; mouse IL-10, 5′-GGTTGCCAAGCCTTATCGGA-3′ and 5′-ACCTGCTCCAC TGCCTTGCT-3′; mouse IL-12p40, 5′-GGAAGCACGGCAGCAGAATA-3′ and 5′-AACTT GAGGGAGAAGTAGGAATGG-3′; and mouse tumor necrosis factor-α (TNF-α), 5′-TCTTCTC ATTCCTGCTTGTGG-3′ and 5′-GGTCTGGGCCATAGAACTGA-3′. The expression of mouse genes was normalized to that of β-actin. The expression levels of mRNA were determined via real-time PCR using the CFX96 PCR system (Bio-Rad, Hercules, CA, USA). The relative gene expression was calculated using the 2-ΔΔCt method.

### 2.11. Protein Extraction and Western Blotting Analysis

After infection with bacteria, adherent cells were washed twice with DPBS and then lysed in a RIPA buffer. After incubation for 1 min, the samples were gently detached from the dishes and then centrifuged at 13,000 rpm for 30 min. The supernatant was collected and stored at −80 °C. The protein concentrations of the lysates were determined using the Bio-Rad Protein Assay Dye Reagent Concentrate (Bio-Rad). The protein was mixed with 5× SDS–Sample buffer (TransLab, Daejeon, Republic of Korea) and denatured by heating to 100 °C for 20 min. 10 to 20 μg of protein was subjected to electrophoresis on 10–12% Bis-Acrylamide gels containing SDS under reducing conditions. Separated proteins were electroblotted onto 0.22 μm polyvinylidene difluoride (PVDF) membranes (Bio-Rad), and blots were blocked with 5% skim milk (*w*/*v*) for 1 h and then washed three times with Tris-buffered saline containing 0.05% Tween 20 (TBS/T). Then, the membranes were incubated overnight at 4 °C with the following antibodies: mouse anti-p-p38 MAPK (#9216S, 1:2000), rabbit anti-p-p44/42 MAPK (#4370S, 1:2000), rabbit anti-p-IKB/alpha (#9246S, 1:1000), mouse anti-p-SAPK/JNK (#4668S, 1:2500; Cell Signaling Technology, Boston, MA, USA), and mouse anti-β actin (#A1978, 1:5000; Sigma–Aldrich, Burlington, MA, USA). Antibody binding was determined using the appropriate secondary antibody coupled with HRP according to the manufacturer’s instructions. Enhanced chemiluminescence was used for the measurement of relevant proteins using the EZ-Western LumiFemto Kit (DoGenBIO).

### 2.12. Seahorse Extracellular Flux Analysis

Oxygen consumption and extracellular acidification rates (OCR and ECAR, respectively) were measured using the Seahorse XFp extracellular flux analyzer (Agilent, Santa Clara, CA). BMDMs were seeded into Seahorse XFp Cell Culture Miniplates (Agilent) at a density of 8 × 10^5^ cells per well and cultured in a 37 °C, 5% CO_2_ incubator overnight. For the latter time point, BMDMs were infected with *Mpgm* strains at an MOI of 2 for 24 h prior to analysis in the Seahorse Analyzer. Prior to measurements, the culture medium was removed and replaced with Seahorse XF DMEM, pH 7.4 (Agilent, catalog #103575–100), and incubated in the absence of CO_2_ for 45 min. For the Mito Stress Test, cells were sequentially treated with oligomycin (1 μM), FCCP (2 μM), and rotenone + antimycin A (0.5 μM). For the glycolysis stress test, cells were sequentially treated with glucose (10 mM), oligomycin (2 μM), and 2-deoxyglucose (50 mM). After analyzing the OCR and ECAR, the cells were lysed, and the protein in each well was quantified using the Bradford assay. All values of OCR and ECAR parameters calculated were normalized to the quantified protein content. Data were analyzed using Wave 2.6.0 software (Agilent Technologies, Santa Clara, CA, USA).

### 2.13. Statistical Analysis

All experiments were repeated at least three times. Data were analyzed using a one-way ANOVA, followed by Tukey’s multiple-comparisons test using GraphPad Prism^®^ 6.01 (GraphPad Software, San Diego, CA, USA). The data in the figures are presented as the mean ± SD. Values of * *p* < 0.05, ** *p* < 0.01 or *** *p* < 0.001, and # *p* < 0.05, ## *p* < 0.01, ### *p* < 0.001 vs. CNT were considered statistically significant.

## 3. Results

### 3.1. M. peregrinum Morphology and Lipid Profile Analysis via Thin-Layer Chromatography

**Figure 1:**
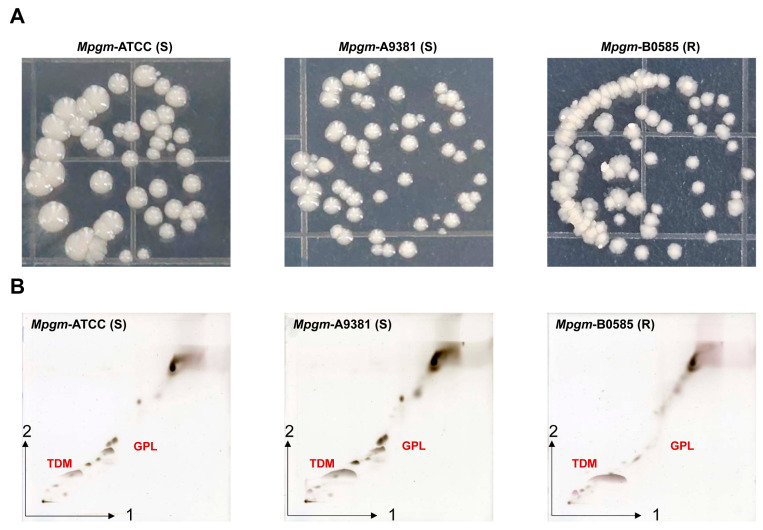
*M. peregrinum* morphology and lipid profile analysis via thin-layer chromatography. (**A**) Colony characteristics of *Mpgm* grown in the 7H10 agar media after 7 days. (**B**) Total lipids were extracted from the *Mpgm* strains ATCC 14467, A9381(S), and B0585(R). Purified trehalose dimyoclate (TDM) and glycopeptidolipids (GPLs) were separated using thin-layer chromatography (TLC). The mobile phase used for TLC was as follows: chloroform/methanol/acetone/acetic acid (90:10:6:1, *v*/*v*/*v*/*v*) and chloroform/methanol/water (90:10:1, *v*/*v*/*v*).

*Mpgm* showed smooth and rough colonies on the 7H10 agar, similar to other NTMs. The reference strain ATCC 14467 exhibited smooth colonies, the clinical strain A9381 displayed smooth colonies, and B0585 showed rough colonies (Figure 1A). Beyond genotypic diversity, *M. abscessus* exhibits considerable phenotypic variability, a factor that is critical to its virulence. Notably, two distinct morphotypes are observed based on the presence or absence of GPLs in the cell wall. Smooth variants possess GPL, whereas rough variants lack this component. This morphological heterogeneity is not unique to *M. abscessus* and is a characteristic seen in various other NTM species [9]. In the TLC data, differences in the lipids of A9381 and B0585 were observed. A9381 exhibited darker bands in the trehalose-6,6-dimycolate (TDM) and GPL regions than B0585, showing a pattern similar to that of the reference smooth colony strain ATCC 14467 (Figure 1B). In the experimental results, we will refer to ATCC 14467 as *Mpgm*-ATCC, A9381 as *Mpgm*-S and B0585 as *Mpgm*-R.

### 3.2. Comparison of the Antimicrobial Susceptibility Results for M. peregrinum

Although the relevance of in vitro drug susceptibility testing is unclear for most NTM species, it is necessary to acknowledge the potential importance of these tests in guiding the management of NTM-related diseases. No prior studies have specifically examined the *Mpgm* strain used in our experiment. Therefore, we referred to antimicrobial susceptibility tests conducted on rapidly growing mycobacteria in other studies [10]. The susceptibility breakpoint, which is the MIC value used to classify strains as susceptible, intermediate, or resistant, indicated that *Mpgm* strains tended to exhibit growth inhibition at low antibiotic concentrations. As shown in Table 1, the MIC values for clarithromycin, moxifloxacin, ciprofloxacin, and tigecycline were found to be ≤0.125 to 0.5 μg/mL. Among these, moxifloxacin exhibited the lowest MIC values. Amikacin, linezolid, clofazimine, imipenem, and meropenem showed MIC values ranging from 1 to 2 μg/mL. Streptomycin, doxycycline, trimethoprim, sulfamethoxazole, and cefoxitin had MIC values ranging from 8 to 32 μg/mL. Notably, there was no significant difference in antibiotic susceptibility between the reference strain and the clinical strains. *Mpgm* was classified as trimethoprim/sulfamethoxazole-resistant (R) with intermediate susceptibility (I) to tobramycin, and susceptible (S) to the remaining antibiotics.

### 3.3. M. peregrinum Is an Intracellular Bacterium

**Figure 2:**
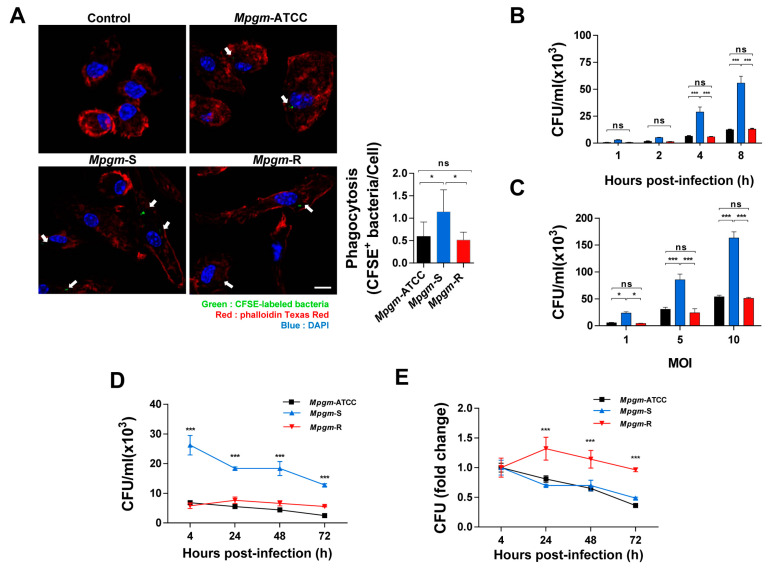
*M. peregrinum* is an intracellular bacterium. (**A**) Intracellular staining of CFSE-coupled *Mpgm* in BMDMs (scale bar, 10 μm). BMDMs were infected with CFSE-coupled bacteria at an MOI of 5 for 4 h. Cortical F-actin was stained using phalloidin–Texas Red, and the nuclei were stained with DAPI. The graph represents the number of CFSE-coupled bacteria per cell. Data are presented as the mean ± SD and are the cumulative results of three experiments (ns: not significant, * *p* < 0.05, *** *p* < 0.001). (**B**) Phagocytosis of BMDMs infected with *Mpgm*. BMDMs were infected with *Mpgm* at an MOI of 1 for 1, 2, 4, or 8 h or (**C**) MOIs of 1, 5, or 10 for 4 h. Noninternalized bacteria were washed twice and BMDMs were lysed with 0.05% Triton X-100 to release intracellular bacteria. The number of bacterial colony-forming units (CFUs) was determined at the specific time point after the removal of the supernatant. (**D**) Changes in the number of *Mpgm* cells (**E**) and fold-change values in BMDMs until 72 h after infection. BMDMs were infected with *Mpgm* at an MOI of 1. At each indicated time point, cells were washed twice and lysed with 0.05% Triton X-100 containing distilled water to release intracellular bacteria.

The capacity to multiply and persist within macrophages is indispensable for the virulence of intracellular pathogens [11]. We confirmed that *Mpgm* is an intracellular pathogen by labeling cells with CFSE and infecting BMDMs. CFSE staining was performed for 2 h, and BMDMs were infected with CFSE-coupled bacteria at an MOI of 5 for 4 h. Cortical F-actin and nuclei were stained with Phalloidin Texas Red and DAPI, respectively, and then analyzed using confocal microscopy. Confocal microscopy analysis showed significantly higher amounts of CFSE-bound bacteria during *Mpgm*-S infections than during *Mpgm*-ATCC and *Mpgm*-R infections (Figure 2A). Additionally, when assessing the number of CFSE-bound bacteria per cell, *Mpgm*-S infection led to approximately three times more bacteria per cell than *Mpgm*-ATCC and *Mpgm*-R infections. *Mpgm*-ATCC and *Mpgm*-R infection led to similar levels of CFSE-bound bacteria (Figure 2A).

Phagocytosis, which is a central process in the elimination of pathogens, is carried out by various immune cells, including neutrophils, macrophages, dendritic cells, and B lymphocytes [12,13]. Macrophages assume an important role in the innate immune system and participate in pathogen identification, phagocytosis, and cytokine production [14,15]. Therefore, we observed the phagocytosis of BMDMs following infection with *Mpgm*-ATCC, S and R. When BMDMs were infected with the three strains for 1, 2, 4, or 8 h at an MOI of 1, phagocytosis increased in *Mpgm*-S-infected BMDMs, while the results of *Mpgm*-R were similar to those of *Mpgm*-ATCC (Figure 2B). Even when infected for 4 h at MOIs of 1, 5, or 10, the number of BMDMs infected with *Mpgm*-S increased in an MOI-dependent manner (Figure 2C). The infection kinetics of *Mpgm*-S showed high activity up to the first 4 h after infection, and the number of intracellular bacteria gradually decreased until 72 h post infection. In the cases of *Mpgm*-ATCC and *Mpgm*-R, infection activity increased up to the first 4 h after infection, but at a lower level than that with *Mpgm*-S, and for both strains, the number of intracellular bacteria did not specifically decrease or increase. However, *Mpgm*-S was still detected at higher levels within cells than the other strains until 72 h post infection (Figure 2D). In addition, interestingly, the amount of the same S-type, represented by *Mpgm*-ATCC and *Mpgm*-S, decreased in macrophages over time, but that of *Mpgm*-R increased slightly until 24 h and then decreased until 72 h (Figure 2E). In summary, *Mpgm* is an intracellular pathogen, and there were differences in the phagocytosis of different strains.

### 3.4. M. peregrinum Does Not Affect the Viability or Cytotoxicity of BMDMs

**Figure 3:**
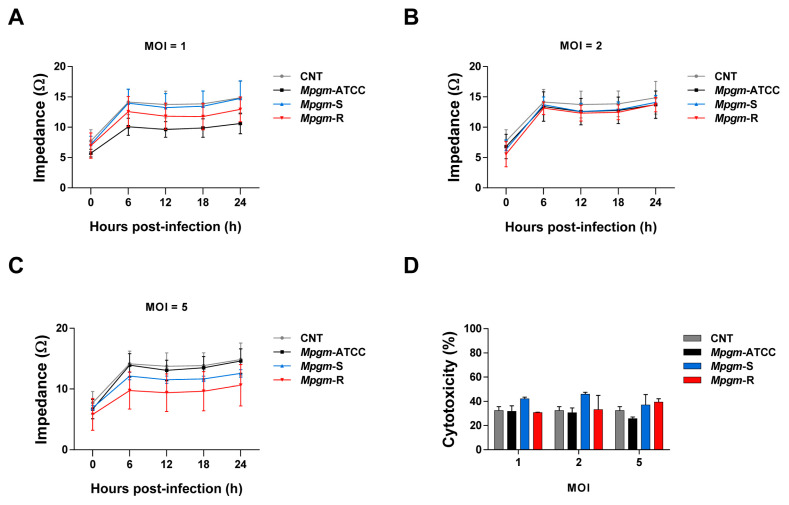
*M. peregrinum* does not affect the viability or cytotoxicity of BMDMs (**A**–**C**). BMDMs were infected with *Mpgm* at MOIs of 1, 2, or 5 for 24 h. Cell viability data were quantified using the Maestro Z analysis program. Cytotoxicity was evaluated by analyzing LDH activity 24 h post infection. (**D**) Supernatants from infected and control BMDMs were collected. LDH release, which is an indicator of cytotoxicity, was quantified using an LDH assay kit. The absorbance was measured at 450 nm.

Intracellular pathogens can lead to infection and death in various cell types, including macrophages [16,17,18]. Host cell death has been observed in numerous instances of bacterial, viral, and parasitic infections, with significant impacts on disease progression [19]. Thus, we determined whether cell death was induced when BMDMs were infected with *Mpgm*. As a result, the *Mpgm*-infected BMDMs displayed similar cell viability profiles to the noninfected controls. There were no significant differences in cell impedance values. We tested at MOIs of 1, 2, or 5 and found no difference between the MOIs (Figure 3A–C). These results indicate that *Mpgm* infection did not adversely affect cell viability. LDH is also one of the methods used to measure cytotoxicity, and LDH release from *Mpgm*-infected BMDMs was comparable to that of noninfected controls. Even with additional experiments, the results were similar (Figure 3D). The absence of significant changes in LDH release indicated that *Mpgm* infection did not lead to increased cellular cytotoxicity. Based on these results, *Mpgm* had little cytotoxicity, and there was no difference between the three strains.

### 3.5. Comparative Analysis of Inflammatory Cytokine Production in BMDMs Infected with M. peregrinum

**Figure 4:**
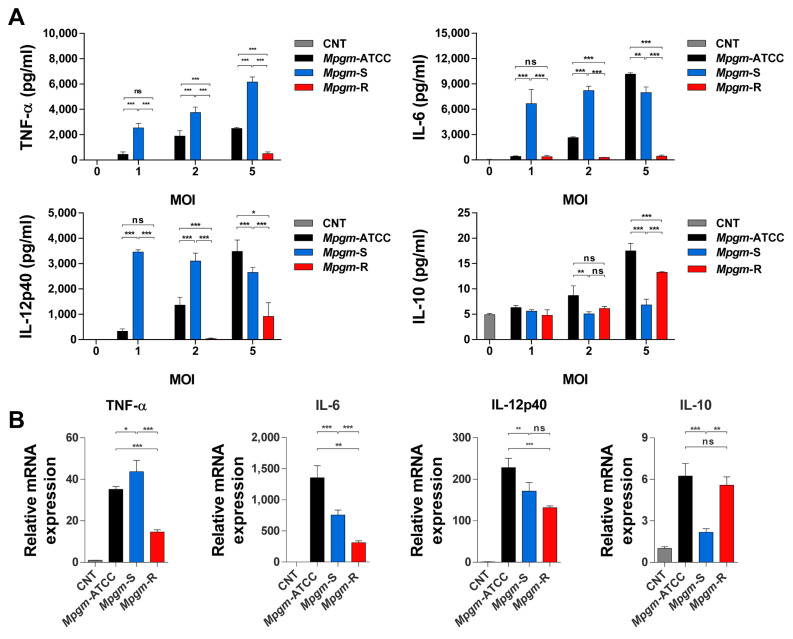
Comparative analysis of inflammatory cytokine production in BMDMs infected with *M. peregrinum.* BMDMs were infected with *Mpgm* strains at MOIs of 1, 2, or 5 for 24 h. Supernatants from infected and control BMDMs were collected. (**A**) The levels of IL-6, IL-10, IL-12p40, and TNF-α were determined via an enzyme-linked immunosorbent assay. The absorbance was read at 450 nm on a microplate reader. (**B**) Total RNA was extracted from control BMDMs and BMDMs infected with *Mpgm* strains at an MOI of 5. cDNA was synthesized, and real-time PCR was performed to analyze the gene expression of cytokines using specific primers. Housekeeping gene expression was used as a reference. Gene expression is represented as the fold-change compared to that of control BMDMs. Data are presented as the mean ± SD and are the cumulative results of three experiments (ns: not significant, * *p* < 0.05, ** *p* < 0.01, *** *p* < 0.001).

During mycobacterial infection, hosts initiate innate and adaptive immune responses to contain the bacteria. These responses involve the production of both proinflammatory and anti-inflammatory cytokines in response to mycobacterial infection [20]. We compared the abilities of ATCC, S-type, and R-type *Mpgm* to induce the secretion of cytokines from BMDMs at MOIs of 1, 2, or 5 at 24 h post infection. As a result, it was confirmed that cytokines were secreted at a higher level when BMDMs were infected with *Mpgm*-S. The levels of, IL-6, and IL-12p40, TNF-α significantly increased upon infection at MOIs of 1, 2 whereas no significant changes were observed in the *Mpgm*-ATCC and *Mpgm*-R infections. When infected at an MOI of 5, BMDMs infected with *Mpgm*-ATCC was higher IL-6 and IL-12p40 levels than BMDMs infected with *Mpgm*-S and *Mpgm*-R. In particular, TNF-α levels increased in an MOI-dependent manner. In contrast, when BMDMs were infected with *Mpgm*-R, the expression of TNF-α was very low. These results indicate that *Mpgm*-S infection induces a proinflammatory environment. The levels of IL-10 were significantly reduced in BMDMs infected with *Mpgm*-S (Figure 4A).

We also performed real-time PCR to assess the relative mRNA levels of inflammatory cytokines in BMDMs infected with *Mpgm* at an MOI of 5 for 24 h. The results were consistent with the cytokine expression trends observed in the ELISA data (Figure 4B). Overall, the cytokine levels caused by infection with *Mpgm*-S tended to be higher than those of *Mpgm*-ATCC and *Mpgm*-R.

### 3.6. M. peregrinum Induces Activation of the MAPK and NF-κB Pathways in BMDMs

Many pathogens trigger signaling pathways, such as those involving MAPK and NF-κB, that are involved in cytokine responses and inflammation [21]. The immune reaction to bacterial invaders, encompassing the intricate interplay between mycobacteria and macrophages, hinges on initiation by MAPKs. These molecular signaling pathways assume pivotal functions in enhancing the body’s defense mechanisms against mycobacterial threats while also orchestrating the synthesis of proinflammatory cytokines [22].

We investigated the cellular immune response to activation of the MAPK and NF-κB pathways in BMDMs during *Mpgm* infection. Cell lysates were harvested at 0, 5, 15, 30, and 60 min, and MAPK and NF-κB phosphorylation was evaluated via immunoblotting. All three strains induced ERK phosphorylation during infection, with *Mpgm*-ATCC and *Mpgm*-S infection leading to persistent phosphorylation through 60 min. Protein levels increased approximately fourfold compared to that of the control. *Mpgm*-R-induced phosphorylation increased for 30 min but was reduced after 60 min.

**Figure 5:**
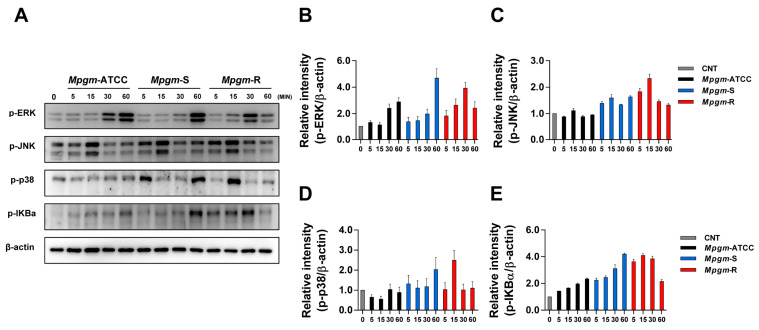
*M. peregrinum* induces activation of the MAPK and NF-κB pathways in BMDMs. Phospho-ERK, JNK, p-38, and IkB-α were activated in *Mpgm*-infected macrophages. (**A**) BMDMs were infected with *Mpgm* at an MOI of 1 for 60 min. Cell lysates were subjected to SDS–PAGE, and immunoblotting analysis was performed using antibodies against phospho-p38 (p-p38), phospho-ERK1/2, phospho-IkBα, phospho-JNK, and β-actin. (**B**–**E**) Each protein band was detected, and relative band intensities were normalized to β-actin. (**B**) The column graphs represent the average relative band intensity with the standard error from three independent experiments. Data are presented as the mean ± SD and are the cumulative results of three experiments.

Additionally, *Mpgm*-R infection of BMDMs induced JNK phosphorylation. In contrast, *Mpgm*-ATCC-infected BMDMs showed similarities to the noninfected control, while *Mpgm*-S and *Mpgm*-R infection caused approximately twofold increases compared to that of the control. Interestingly, the phosphorylation of p38 occurred at different time points, with *Mpgm*-S inducing phosphorylation at 60 min, whereas *Mpgm*-R induced phosphorylation at 15 min, and *Mpgm*-ATCC showed similarities to the noninfected control. Phosphorylation of IKBα was triggered by all strains, with *Mpgm*-S showing increased phosphorylation at 60 min. In contrast, *Mpgm*-R maintained phosphorylation at a similar level for 30 min (Figure 5A–E). In our data, p38 phosphorylation persisted for up to 60 min upon *Mpgm*-S infection, and IKBα phosphorylation was also the highest at 60 min. These data suggest that *Mpgm*-S induces significantly greater MAPK and NF-κB activation than the *Mpgm*-ATCC and *Mpgm*-R strains.

### 3.7. Metabolic Profile of BMDMs Infected with M. peregrinum

**Figure 6:**
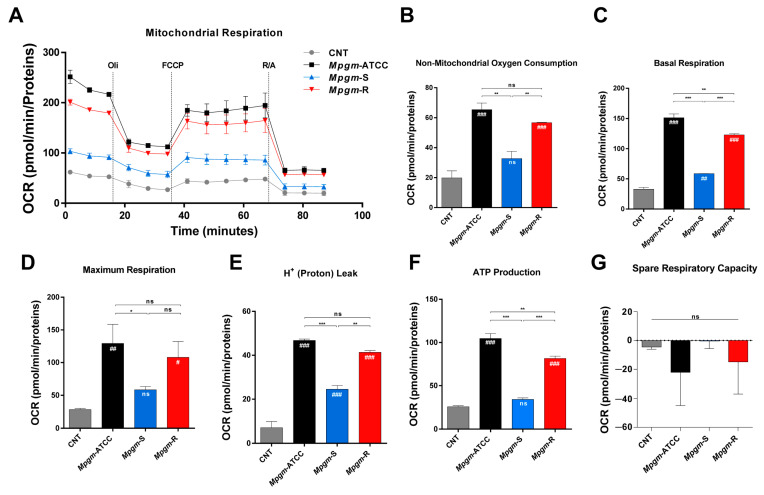
Respiratory profiles and parameters of BMDMs infected with *M. peregrinum.* (**A**) Real-time respiratory profiles (OCR) of BMDMs infected with *Mpgm* at an MOI of 2 for 24 h. (**B**) Quantification of nonmitochondrial oxygen consumption, (**C**) basal respiration, (**D**) maximal respiration, (**E**) H^+^ (proton) leak, (**F**) ATP production, and (**G**) spare respiratory capacity. The real-time respiratory profiles and parameters are representative of three independent experiments. Data in B–G were analyzed via a one-way ANOVA with Tukey’s multiple-comparisons test. Data are presented as the mean ± SD and are the cumulative results of three experiments (ns: not significant, * *p* < 0.05, ** *p* < 0.01, *** *p* < 0.001, # *p* < 0.05, ## *p* < 0.01, ### *p* < 0.001 vs. CNT).

**Figure 7:**
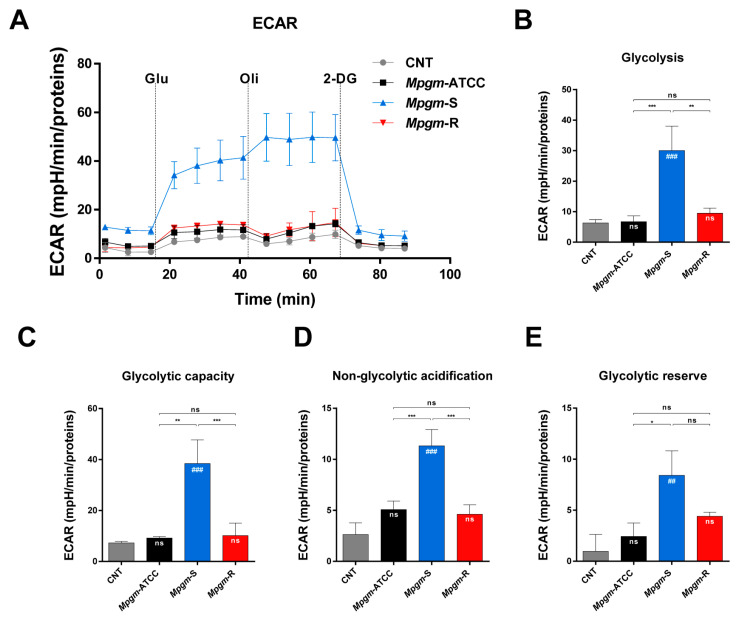
Extracellular acidification profiles and glycolytic parameters of BMDMs infected with *M. peregrinum.* (**A**) Real-time extracellular acidification (ECAR) of BMDMs infected with *Mpgm* at an MOI of 2 for 24 h. (**B**) Quantification of glycolysis activity, (**C**) glycolytic capacity, (**D**) nonglycolytic acidification, and (**E**) glycolytic reserve. The real-time extracellular acidification profiles and parameters are representative of three independent experiments. Data in B–E were analyzed via a one-way ANOVA with Tukey’s multiple comparisons test. Data are presented as the mean ± SD and are the cumulative results of three experiments (ns: not significant, * *p* < 0.05, ** *p* < 0.01, *** *p* < 0.001, ## *p* < 0.01, ### *p* < 0.001 vs. CNT).

Several reports have shown that intracellular bacterial pathogens can disrupt and/or reprogram the metabolic health of macrophages for their own benefit, thereby facilitating the establishment of successful infections [23,24]. Consequently, an analysis of the shifts in metabolic processes within macrophages during infection can contribute to our understanding of how *Mpgm* leads to disease. We examined the impact of *Mpgm* infection on the oxidative phosphorylation (OXPHOS) of BMDMs using a cell mito stress test kit (Figure 6), and analyzed the effect of infection on glycolysis in BMDMs utilizing a glycolysis stress test kit (Figure 7). First, we sequentially treated *Mpgm*-infected BMDMs with oligomycin, FCCP, and rotenone/antimycin A and measured the oxygen consumption rate (Figure 6A). Figure 6 shows that infection with *Mpgm*-ATCC and *Mpgm*-R significantly increased the following respiratory parameters in BMDMs: nonmitochondrial respiration parameters, basal respiration, maximum respiration, H+ (proton) leakage, and ATP production. In contrast, the respiratory parameters in *Mpgm*-*S*-infected BMDMs were comparable to those in the control (Figure 6B–G). Next, we sequentially treated *Mpgm*-infected BMDMs with D-glucose, oligomycin, and 2-deoxyglucose and measured the extracellular acidification rate (Figure 7A). Contrary to previous results in terms of respiratory parameters, glycolysis activity, glycolytic capacity, nonglycolytic acidification, and glycolytic reserve were significantly increased in only *Mpgm*-*S*-infected BMDMs. In contrast, the parameters of glycolytic functions in *Mpgm*-ATCC- and *Mpgm*-R-infected BMDMs were comparable to those of the control (Figure 7B–E). In summary, we observed significant differences in glycolytic and respiratory functions between the control group and BMDMs infected with *Mpgm*. Specifically, *Mpgm*-ATCC and *Mpgm*-R infections notably increased respiratory parameters, while *Mpgm*-S infection significantly promoted glycolytic functions.

## 4. Discussion

*Mycobacterium peregrinum* (*Mpgm*) belongs to the category of rapidly growing nontuberculous mycobacteria (RGMs) and is primarily associated with pathogenicity in lung, skin, soft tissue, and bone infections [5]. *Mpgm* infections have been reported in small numbers and can cause chronic lung disease, sternal wound infections, and skin disease [5]. Some of the newer cases have included nonreversible infections, bacteremia, and fatal pneumonia [25,26,27]. The treatment approach for rapidly growing mycobacteria can vary depending on specific disease presentation. In minor cases, single drug therapy may be appropriate, while disseminated skin and lung diseases may require combination treatment with antibiotics [5]. Knowledge of the susceptibility patterns of *Mpgm* to many antibiotics alone and in combination would be of great interest, especially when considering the limited information available about potential treatments [28].

In this study, we focused on confirming the characteristics of *Mpgm* and basic research on changes in the immune response and metabolic reprogramming that occur when BMDMs are infected with *Mpgm*. *Mpgm*-ATCC and *Mpgm*-S grew as smooth colonies on Middlebrook 7H10 agar, whereas *Mpgm*-R grew as rough colonies. Differences in colony morphology depend on the presence of surface-associated GPLs in S and not in R *Mpgm* strains [29,30]. Similar to previous studies, our TLC results show that the GPL region appears dark in *Mpgm*-*S* but not in *Mpgm*-R.

RGMs pose a significant clinical challenge due to their capacity to instigate a spectrum of infections that affect various body regions, including the lungs, skin, and soft tissues [31]. Due to the diverse drug susceptibility profiles exhibited by different RGM isolates, treatment strategies are based on each specific strain. Consequently, there are still isolated cases where the optimal treatment approach remains uncertain [32]. Therefore, we evaluated antimicrobial activity, and the results showed that all strains were resistant to doxycycline and trimethoprim/sulfamethoxazole, intermediately resistant to tobramycin, and susceptible to other antibiotics, including clarithromycin and amikacin.

Numerous studies have identified a correlation between colony appearance and virulence, with strains with rough morphologies typically exhibiting a higher level of virulence than those with smooth morphologies [33]. However, our data showed that the intracellular survival of *Mpgm*-S in macrophages was higher than that of *Mpgm*-ATCC and *Mpgm*-R. *Mpgm*-S had increased intracellular survival in a time-dependent and MOI-dependent manner, with a significant increase compared to that of *Mpgm*-ATCC and *Mpgm*-R. Following infection with the three strains and observation for 72 h, *Mpgm*-S levels tended to decrease after 4 h, but the intracellular survival rate was higher than that of *Mpgm*-R. *Mpgm*-R showed no significant change in the number of bacteria 72 h after infection. Infection with NTM does not always increase the number of intracellular bacteria and this number may vary depending on experimental conditions [34,35].

We also observed no significant difference in cell viability following infection. The same results were obtained for cytotoxicity as with cell viability. This result is consistent with previous studies involving other mycobacterial species. However, compared to other studies, cell viability and toxicity were not evaluated for a longer period.

TNF-α is a cytokine released upon immune system activation. Mainly secreted by macrophages, TNF-α secretion can also occur via lymphocytes, mast cells, endothelial cells, and fibroblasts [36]. Because most cells are responsive to TNF-α, and TNF-α is considered a major proinflammatory mediator. Regarding host immunity, the production of IL-12 is an important factor for the establishment of Th1 immunity and effective defense against intracellular pathogens [37]. IL-6 is an important cytokine associated with inflammatory diseases. The accumulation of IL-6 occurs through interactions with other cytokines, such as TNF-α [38]. Previous studies have demonstrated that IL-6 can promote the intracellular proliferation of mycobacteria within monocytes [39]. We found that *Mpgm*-S infection significantly increased the expression of the proinflammatory cytokines IL-6, IL-12p40, and TNF-α. In particular, the expression of TNF-α was upregulated in an MOI-dependent manner. Furthermore, IL-10 expression was upregulated during *Mpgm*-ATCC and *Mpgm*-R infection, and the same result was obtained via RT–PCR.

Many pathogens activate signaling pathways, such as the MAPK and NF-κB pathways, which play crucial roles in triggering cytokine responses and promoting inflammation [21]. The MAPK family includes the following three primary serine–threonine protein kinases: p38, ERK, and c-Jun NH2-terminal kinase. Several investigations have compared MAPK signaling levels in macrophages upon mycobacterial infection. These studies indicate that the activation levels and temporal patterns of p38 and ERK1/2 differ based on the specific mycobacterial species [22,40].

Numerous investigations have revealed a correlation between varying levels and timing of MAPK activation and distinctions in colony morphology among closely related bacterial strains. For example, in the case of *M. abscessus*, colonies frequently exhibit a rough morphology, which is attributed to a deficiency in GPLs on the bacterial surface. This characteristic induces these strains to be more pathogenic, resulting in the activation of MAPK signaling and consequently increased inflammatory responses compared to bacteria with smooth colonies [40,41]. Notably, unlike in previous studies, our data showed that *Mpgm*-S is more toxic than *Mpgm*-ATCC and *Mpgm*-R. Regarding signaling pathways, both *Mpgm*-S and *Mpgm*-R induced NF-κB nuclear translocation and phosphorylated markers of the MAPK pathways. Moreover, *Mpgm*-S mycobacteria induced a higher degree of MAPK phosphorylation and IκB degradation than their *Mpgm*-ATCC and *Mpgm*-R counterparts, providing compelling evidence that *Mpgm*-S mycobacteria efficiently and rapidly engage macrophages via the MAPK and NF-κB pathways, consequently promoting the upregulation of proinflammatory cytokine expression.

During macrophage–intracellular pathogen interactions, both macrophages and intracellular pathogens compete for the limited pool of nutrients needed for cellular catabolism [42,43]. In this context, intracellular pathogens have developed many strategies to manipulate the intracellular environment and render it beneficial for their own purpose, notably by modifying mitochondrial integrity and function to influence energy production, metabolism processes, and immune signaling pathways [24]. Nonetheless, the mechanism by which NTM manipulates macrophage energy metabolism to facilitate survival has not been fully elucidated. Here, we used extracellular flux analysis to explore the change in the energy metabolism of BMDMs infected with three strains of *Mpgm*. *Mpgm* infection triggered a metabolic shift in infected BMDMs toward a higher energy state. This shift was observed in *Mpgm*-ATCC- and *Mpgm*-R-infected BMDMs, which exhibited increased OXPHOS, while *Mpgm*-S-infected BMDMs showed enhanced glycolysis. Macrophages, which were characterized by a proinflammatory phenotype that includes increased phagocytosis and the production of proinflammatory cytokines, are known to depend more on glycolysis and less on OXPHOS for energy generation [44].

Our results revealed increased levels of proinflammatory cytokines (Figure 4), increased phagocytosis (Figure 2B–C), and reduced intracellular bacterial survival (Figure 2D) in *Mpgm*-S-infected macrophages; these results align with the findings, indicating that proinflammatory macrophages rely on glycolysis rather than OXPHOS for energy production. Furthermore, a recent investigation revealed that *M. tuberculosis* infection triggers a metabolic program reminiscent of the Warburg effect in human primary macrophages. This program is characterized by a reduction in OXPHOS, a surge in glucose uptake and glycolysis, and a reconfiguration of glycolytic intermediates within the macrophage. In contrast, infection with the vaccine strain BCG increased respiratory parameters of human primary macrophages, which is characteristic of a healthy inflammatory response [45].

In our studies, infection with *Mpgm*-ATCC and *Mpgm*-R significantly enhanced OXPHOS in BMDMs, whereas *Mpgm*-S infection notably promoted glycolysis. These data suggest that *Mpgm*-S has greater pathogenicity in macrophages than *Mpgm*-R, as has been observed in the response to *M. tuberculosis* and BCG.

## 5. Conclusions

In conclusion, these results suggest that *Mpgm*-S is more pathogenic and induces a more robust immune response than *Mpgm*-ATCC and *Mpgm*-R. This response appears different from those of other NTM species, such as *M. abscessus* and the *M. avium* complex. In addition, *Mpgm*-S infection affects the immune response of mouse macrophages more than infection by *Mpgm*-R. This study is significant, as it is the first to report on the immune response of macrophages to *Mpgm* infection. These findings may serve as a foundation for future investigations of the pathogenesis of *Mpgm*.

## Figures and Tables

**Table 1:** Comparison of antimicrobial susceptibility results of *M. peregrinum* strains.

MIC (μg/mL)						
Antibiotic	*Mpgm*-ATCC	Interpretation	*Mpgm*-S	Interpretation	*Mpgm*-R	Interpretation
Clarithromycin	≤0.5	S	≤0.5	S	≤0.5	S
Amikacin	≤2	S	≤2	S	≤2	S
Moxifloxacin	≤0.125	S	≤0.125	S	≤0.125	S
Linezolid	2	S	2	S	4	S
Streptomycin	32	–	8	–	4	–
Ciprofloxacin	≤0.25	S	≤0.25	S	≤0.25	S
Doxycycline	≥16	R	≥16	R	16	R
Clofazimine	1	–	0.5	–	≤0.25	–
Trimethoprim	32	R	8	R	4	R
Sulfamethoxazole	608	R	152	R	76	R
Cefoxitin	8	S	16	S	8	S
Imipenem	≤1	S	2	S	≤1	S
Meropenem	≤1	S	4	S	2	S
Tobramycin	4	I	4	I	4	I
Tigecycline	≤0.125	–	≤0.125	–	≤0.125	–

Antimicrobial susceptibility testing (AST) was performed using a panel of commonly used antimicrobial agents, including the antibiotics clarithromycin and amikacin moxifloxacin. The minimum inhibitory concentrations (MICs) of these antibiotics against *Mpgm* isolates were determined using established protocols.

## Data Availability

Any data or material that support the findings of this study can be made available by the corresponding author upon request.
